# Decision-making of construction workers' waste reduction behavior: a study based on Cost-Benefit Theory and Cumulative Prospect Theory

**DOI:** 10.3389/fpsyg.2025.1557736

**Published:** 2025-03-20

**Authors:** Shuitai Xu, Yuhui Zhou, Simei Xu, Jingkuang Liu, Qirong Chen, Fei Xue, Wenxing Zhu

**Affiliations:** ^1^School of Economics and Management, Jiangxi University of Science and Technology, Ganzhou, China; ^2^Research Center of Mining Development, Jiangxi University of Science and Technology, Ganzhou, China; ^3^School of Management, Guangzhou University, Guangzhou, China; ^4^School of Civil Engineering, Chongqing University, Chongqing, China

**Keywords:** construction waste, waste reduction behavior, decision-making, Cost-Benefit Theory, Cumulative Prospect Theory

## Abstract

The rapid advancement of industrialization and urbanization has led to a significant generation of construction waste, causing serious resource wastage and environmental pollution. To promote the sustainable development of the construction industry, this study integrates Cost-Benefit Theory and Cumulative Prospect Theory to develop a decision-making model for construction workers' waste reduction behavior (CWWRB), examining the decision-making process under the influence of self-interested motivations and cognitive biases among construction workers. This study, using a construction project in Shenzhen, China, as a case study, assigns variable values and designs management scenarios based on field interview data to simulate the impact of management measures on the decision-making of CWWRB, and the results indicate that: (1) Increasing the negative incentive level significantly promotes the decision-making of CWWRB, but a diminishing marginal effect is observed. (2) Optimizing the atmosphere for waste reduction exerts a lagging guiding effect on the decision-making of CWWRB. (3) Combined measures can compensate for the diminishing marginal effect and lagging effect of single measures, thereby enhancing management effectiveness. The findings not only enrich the theoretical framework for construction waste management but also provide theoretical support for formulating effective management strategies.

## 1 Introduction

The rapid advancement of the global economy, coupled with industrialization and urbanization, has resulted in an annual production of construction waste exceeding 10 billion tons, which constitutes approximately 40% of the total volume of urban solid waste worldwide (Wu et al., 2019; Lin et al., 2024a). Construction waste has caused serious resource wastage and negative environmental impacts, conflicting with the United Nations' development goal of “sustainable cities and communities” (United Nations, 2021; Lin et al., 2024b). In response to the above challenges, governments worldwide have actively implemented measures to manage construction waste. For example, since 2008, the European Union has implemented a waste management hierarchy of “prevention > preparation for reuse > recycling > other recovery > disposal,” significantly increasing the recycling rate of construction waste to 90% (Zhang et al., 2022). In 2010, Vietnam introduced the LOTUS Green Building Certification System, requiring contractors to incorporate construction waste management into construction plans (Lockrey et al., 2016). Since 2021, China has implemented tax relief policies for enterprises engaged in construction waste utilization, boosting the recycling rate to 40% (Ministry of Finance of the People's Republic of China, 2021; Lin et al., 2024a). However, most countries still dispose of construction waste by direct landfilling or dumping, which not only pollutes the air, soil, and water but also leads to land occupation and ecological damage (Arhoun et al., 2022; Zhang et al., 2022; Yu et al., 2024). Therefore, it is urgent and necessary to promote source reduction of construction waste to minimize resource wastage and negative environmental impacts.

The effectiveness of construction waste source reduction is significantly influenced by the behaviors of construction workers during the construction phase (Teo and Loosemore, 2001; Jin et al., 2019). Although existing studies have explored the driving factors for the decision-making of construction workers' waste reduction behavior (CWWRB), there is a lack of focus on the decision-making process (Bakshan et al., 2017). Therefore, this study investigates the mechanisms triggering the decision-making of CWWRB, aiming to provide a scientific basis for formulating management strategies, thereby promoting the high-quality development of the construction industry.

Construction workers, as decision makers who seek economic rewards, particularly value the impact of decision-making outcomes on maximizing personal economic interests (Yuan and Li, 2018). Specifically, factors such as wage income, physical exertion, additional rewards, and losses from violations constitute the key considerations in construction workers' decision-making regarding construction waste reduction. Given that Cost-Benefit Theory, as an effective tool for guiding optimal economic decisions, is based on the “Homo Economicus Hypothesis” and emphasizes that decision-makers maximize interests by comprehensively evaluating the costs and benefits of alternatives, whose core viewpoint is highly compatible with the decision-making mode of construction workers (Sommerville et al., 2018). Therefore, this study adopts Cost-Benefit Theory to investigate the rational choices of construction workers influenced by self-interested motivations. However, in real decision-making scenarios, construction workers are subject to multiple constraints such as environmental uncertainty, information asymmetry, and cognitive limitations, inevitably resulting in cognitive biases such as reference dependence, loss aversion, and probability weighting, which make rational judgments difficult (Curtis and Curtis, 2024). Cost-Benefit Theory has limited explanatory power for the decision-making of bounded rational individuals, while Cumulative Prospect Theory, by introducing reference points, value functions, and weighting functions, offers a significant advantage in accurately portraying the decision-making under cognitive biases (Cao et al., 2024). Therefore, this study integrates Cumulative Prospect Theory with Cost-Benefit Theory to explore construction workers' decision-making process under the combined effect of self-interested motivations and cognitive biases. Moreover, management measures significantly influence construction workers' decisions (Hao et al., 2022). Therefore, this study explores how the decision-making responds to changes in management scenarios to provide scientifically improved strategies for construction waste management in practice.

**Figure 1:**
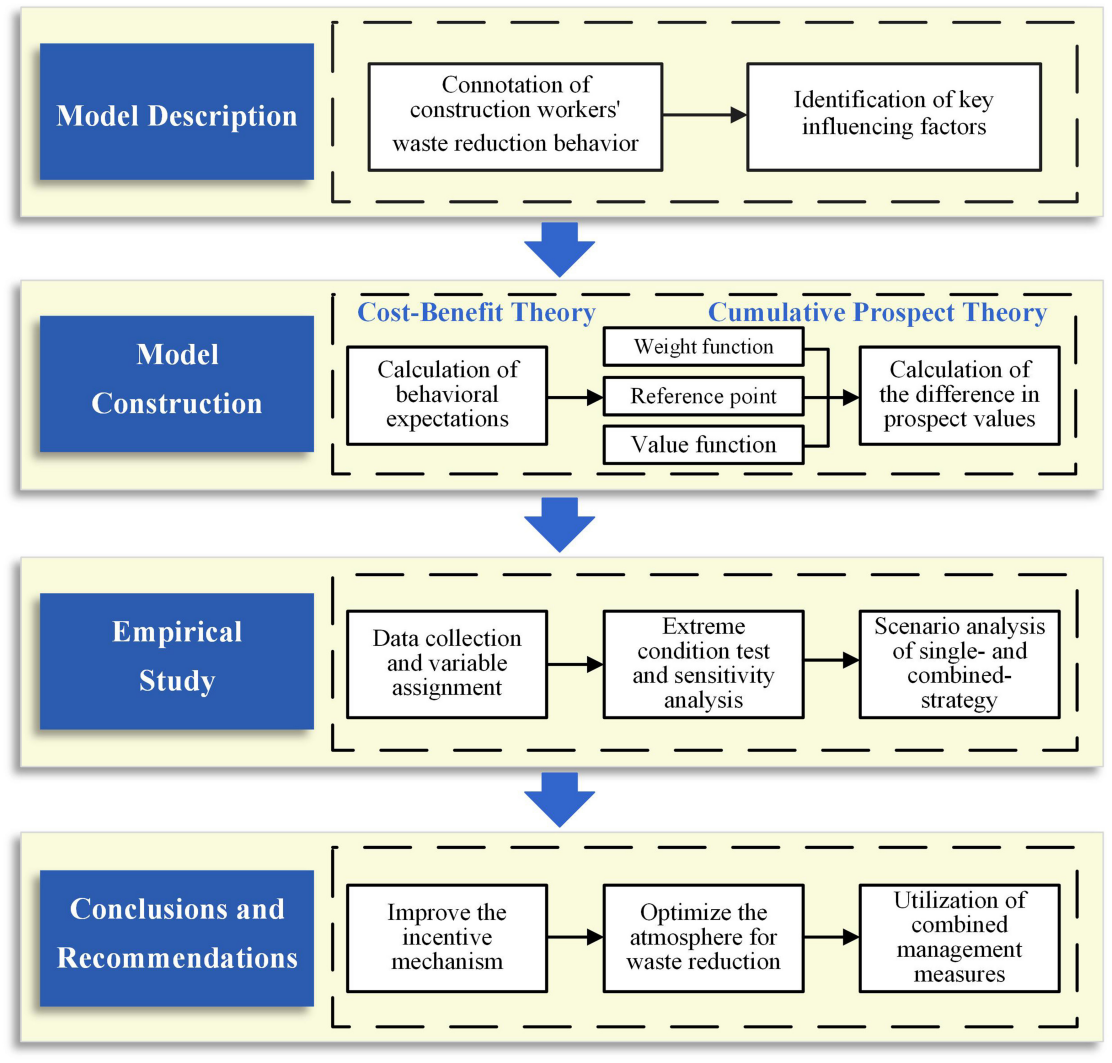
Research frame diagram.

In summary, based on Cost-Benefit Theory and Cumulative Prospect Theory, the study aims to address the following questions: (1) How do construction workers' self-interested motivations affect the decision-making of CWWRB? (2) How do construction workers' cognitive biases influence the decision-making of CWWRB? (3) What specific effects do changes in key management measures have on the decision-making of CWWRB? To explore the above questions, this study constructs a research framework as shown in Figure 1, which demonstrates the logical structure of this study. Based on this, the primary innovations and contributions of this study include: (1) By integrating Cost-Benefit Theory and Cumulative Prospect Theory, this study investigates the formation mechanism of the decision-making of CWWRB under the influence of self-interested motivations and cognitive biases. (2) This study constructs various management scenarios to simulate the dynamic response of the decision-making of CWWRB to fluctuations in key management factors. (3) Based on the simulation results, this study provides specific recommendations to guide CWWRB, offering a reference for the formulation of management strategies for construction waste reduction in practice.

## 2 Literature review

### 2.1 Decision-making of construction workers' waste reduction behavior (CWWRB)

Construction workers' behavior significantly impacts construction waste reduction management (Jin et al., 2019), which has attracted widespread academic attention to the decision-making of CWWRB (Bakshan et al., 2017). Current studies primarily focus on the drivers of the decision-making of CWWRB. For example, Hao et al. (2022) used multivariate linear regression analysis to identify the key factors influencing the decision-making of CWWRB. Additionally, Suciati et al. (2018) confirmed the crucial guiding role of management support factors in the decision-making of CWWRB. Furthermore, Yuan and Li (2018), based on the Theory of Planned Behavior (TPB), found that the decision-making of CWWRB is mainly driven by self-interest. However, existing studies have paid insufficient attention to the formation mechanism of the decision-making of CWWRB, which limits the practical application of research findings.

Currently, research examining behavioral decision-making in waste management primarily employs TPB (Li et al., 2018; Jain et al., 2020), statistical methods (Li et al., 2022), Cost-Benefit Theory (Lin et al., 2020), and Cumulative Prospect Theory (Su and Sun, 2023). TPB and statistical methods are commonly employed to analyze the factors that drive decisions. For instance, Yang et al. (2020) based on TPB found that skills training and resource constraints significantly influence the probability that construction workers reduce construction waste. An analysis using Bayesian networks conducted by Bakshan et al. (2017) revealed the roles of practical experience and social pressure in motivating the decision-making of CWWRB. Additionally, while Cost-Benefit Theory has been used to analyze the decision-making mechanisms of pro-environmental behaviors of benefit-driven individuals (Li et al., 2023), it does not account for the impact of cognitive biases, thereby limiting the accuracy of behavior prediction (Ebrahimigharehbaghi et al., 2022). To deal with the above shortcomings, Geng et al. (2023) used Cumulative Prospect Theory to address the cognitive biases in individual decision-making within uncertain and risk environments. Further research by Yang and Wang (2021) has confirmed that integrating Cumulative Prospect Theory with Cost-Benefit Theory effectively reveals the intrinsic formation mechanism of decision-making among construction workers. Consequently, this study blends Cost-Benefit Theory with Cumulative Prospect Theory, exploring the decision-making process of CWWRB influenced by self-interested motivations and cognitive biases.

### 2.2 Construction waste reduction management measures

The implementation of management measures can significantly reduce the production of construction waste, attracting considerable attention from both governments and enterprises (Yuan and Wang, 2014). Hao et al. (2019) evaluated the economic benefits of landfill charging measures, aiming to set reasonable charging rates to encourage contractors to perform on-site sorting. Further, Wang et al. (2021) suggested that a combination of guided and mandatory measures could improve management efficiency. Lu et al. (2021) showed that applying prefabricated components can reduce construction waste by 15.38%. Eze et al. (2024) introduced Building Information Modeling to reduce construction waste caused by design flaws, unrefined construction planning, and contractual disputes.

Management measures significantly influence construction workers' decisions (Liu et al., 2022). Yuan et al. (2012) pointed out that training is an effective measure to hinder construction workers' waste non-reduction behavior (CWWNRB). Yuan and Li (2018) further discovered that incentive measures help construction workers translate the willingness to reduce waste into actual actions. Nevertheless, existing studies primarily focus on the impact of management measures on reduction targets and managers' decisions (Hao et al., 2019; Lu et al., 2021), with insufficient exploration of how construction workers' decision-making specifically responds to these measures. Therefore, this study constructs various management scenarios to empirically analyze the decision-making of CWWRB under different management constraints.

### 2.3 Literature critique

In summary, significant progress has been made in construction waste reduction research, yet two main deficiencies remain. First, most existing studies have used TPB and statistical methods to explore the drivers of the decision-making of CWWRB, but few have delved into the intrinsic formation mechanism of decision-making. In particular, it is unclear how self-interested motivations and cognitive biases, as key factors, specifically affect the decision-making of CWWRB (Yuan and Li, 2018; Yang and Wang, 2021). Second, existing studies focus on evaluating the effectiveness of management measures at the project and managerial levels, with insufficient research on the specific impact on construction workers' decision-making. Therefore, this study, integrating Cost-Benefit Theory with Cumulative Prospect Theory, reveals the decision-making processes of CWWRB influenced by self-interested motivations and cognitive biases. Moreover, this study investigates construction workers' decision-making in different management scenarios, to provide theoretical support for improving construction waste management efficiency.

## 3 Model construction

### 3.1 Factors identification and behavioral decision-making analysis from the perspective of Cost-Benefit Theory

Cost-Benefit Theory, grounded in the “Homo Economicus Hypothesis,” emphasizes that decision-makers can comprehensively identify and compare the expected costs and benefits of alternative options through rational thinking, and base the decisions on the principle of achieving the highest economic return with the minimum economic input. The origins of Cost-Benefit Theory can be traced back to the nineteenth century, with the “Principle of Cost-Benefit in Public Utilities” formulated by Dupuit (1844) in public project evaluation and regarded as the early form of the theory. As economic theory has evolved, Cost-Benefit Theory has matured and its application has expanded to fields such as business management (Zhang and Chen, 2021), public services and social security (Roberts et al., 2005), and environmental protection (Torres et al., 2023), serving as a crucial analytical tool for addressing complex socio-economic issues. Furthermore, Becker (1976) has demonstrated the applicability of Cost-Benefit Theory in behavioral sciences, with research indicating that individuals generally adhere to the principle of maximizing personal self-interest in decisions, with anticipated costs and benefits acting as key determinants.

Based on the core tenets of Cost-Benefit Theory, the decision-making logic of CWWRB can be reasonably explained. As decision-makers focused on earning wages, construction workers exhibit rational characteristics of avoiding harms and pursuing self-interest maximization in decision making, aligning closely with the core hypothesis of Cost-Benefit Theory. Specifically, additional rewards can incentivize construction workers to engage in waste reduction, while additional costs and risks significantly deter the willingness to reduce waste. Therefore, this study aims to utilize Cost-Benefit Theory to explore the rational choices of construction workers.

From the perspective of Cost-Benefit Theory, construction workers decide whether to take practical actions to reduce construction waste, based on absolute economic benefits (Hao et al., 2022). This study identifies and analyzes the key factors influencing the decision-making of CWWRB, including cost-benefit factors and the environmental factors. Benefit factors refer to the economic interests obtained by construction workers for labor, including work income of construction workers (WI) and positive incentive level (PIL). Cost factors refer to the price paid by construction workers for obtaining benefits, including working cost (WC), the additional working cost for CWWRB (AWC), the negative incentive level (NIL), and the wasteful education and training effects of CWWNRB (WETE). Specifically, additional benefits, such as PIL, stimulate CWWRB, whereas AWC hinders CWWRB. Additionally, WETE and NIL, identified as extra losses and risk costs, significantly constrain CWWNRB.

Environmental factors, such as interactions among construction workers (Yang and Wang, 2021), production pressure (Lingard et al., 2000; Tam, 2008), skill training (Li et al., 2022), regulatory intensity (Yang et al., 2020), incentive mechanisms (Udawatta et al., 2015), and on-site waste reduction atmosphere (Essl et al., 2021), jointly influence the probability that construction workers perform waste reduction behavior (P), as detailed in Equation 1. Specifically, the collaborative work mode within construction teams facilitates the dissemination of CWWRB, wherein reminders from colleagues can effectively stimulate construction workers' willingness to reduce construction waste (Shan, 2022). However, high production pressure and the absence of skill training hinder the transformation of the willingness to reduce waste into practical actions (Kulatunga et al., 2006; Xu et al., 2023). Conversely, research by Udawatta et al. (2015) confirmed that the combination of incentives and on-site regulatory measures significantly promotes CWWRB. Furthermore, Shan (2022) and Yuan et al. (2012) indicate that a positive waste reduction atmosphere enhances construction workers' acceptance of management norms, under the dual influence of production pressure and peer reminders, leading construction workers to comply with construction waste management regulations and implement waste reduction behaviors voluntarily.

Based on the above factors, this study calculates the behavioral expectations under different scenarios, as shown in Equation 1. In Equation 1, E_rd_ is the expectation of CWWRB being discovered, E_rnd_ represents the expectation of CWWRB being not discovered, E_nrd_ refers to the expectation of CWWNRB being discovered, E_nrnd_ denotes the expectation of CWWNRB being not discovered, AE is the average expectation of behavioral decision-making, P characterizes the probability that construction workers perform waste reduction behavior, and P_0_ represents the initial value of P. A detailed description of the variables involved in this study is shown in Table 1.


(1) $$\begin{array}{l} \;E_{rd}=WI-WC-AWC+PIL\\ \;E_{rnd}=WI-WC-AWC\\ \;E_{nrd}=WI-WC-WETE-NIL\\ \;E_{nrnd}=WI-WC-WETE\\ \;AE=P\times MP\times E_{rd}+P\times \left(1-MP\right)\\ \times E_{rnd}+\left(1-P\right)\times MP\times E_{nrd}+\left(1-P\right)\times \left(1-MP\right)\times E_{nrnd}\\ \;P=P_{0}\times IMRP\times IMIP\times IIP\times IREET\times IPP \end{array}$$

**Table 1:** Descriptions on the variables quoted in this study.

**Variables**	**Description**	**Unit**
AWC	Additional working cost for CWWRB	CNY
AE	Average expectation of behavioral decision-making	CNY
AEP	Average expectation of behavioral decision-making on the previous day	CNY
AWR	Atmosphere for waste reduction	1
WC	Working cost	CNY
DPV	Difference in prospect value between CWWRB and CWWNRB	CNY
E_nrd_	Expectation of CWWNRB being discovered	CNY
E_nrnd_	Expectation of CWWNRB being not discovered	CNY
E_rd_	Expectation of CWWRB being discovered	CNY
E_rnd_	Expectation of CWWRB being not discovered	CNY
IFEET	Impact of full effects of education and training on the probability of CWWRB	1
IMRP	Impact of mutual reminders by construction workers on the probability of CWWRB	1
IMIP	Impact of manager intervention on the probability of CWWRB	1
IIP	Impact of incentive on the probability of CWWRB	1
IREET	Impact of remaining effects of education and training on the probability of CWWRB	1
IPP	Impact of production pressure on the probability of CWWRB	1
MP	Probability that CWWRB or CWWNRB is detected by managers	1
NIL	Negative incentive level	CNY
P	Probability that construction workers perform waste reduction behavior	1
PCWR	Probability of construction workers reminding others	1
PIL	Positive incentive level	CNY
PP	Production pressure	1
PVNRB	Prospect value of construction workers' CWWNRB	CNY
PVRB	Prospect value of construction workers' CWWRB	CNY
REET	Remaining effects of education and training	CNY
WETE	Wasteful education and training effects of CWWNRB	CNY
WI	Work income of construction workers	CNY

### 3.2 Behavioral decision-making analysis from the dual perspectives of Cost-Benefit Theory and Cumulative Prospect Theory

Cumulative Prospect Theory, proposed by Tversky and Kahneman (1992), is an axiomatic modification and extension of Prospect Theory, aiming to provide a theoretical framework for studying the decision-making of bounded rational individuals in uncertainty and risky environments. Cumulative Prospect Theory challenges the applicability of traditional Expected Utility Theory in real-world decision-making scenarios, emphasizes the reference-dependent nature of decision-makers, permits asymmetric risk attitudes toward losses and gains, and reveals cognitive distortions in probability perception of low-probability and high-probability events, which significantly the enhances explanatory power of the theory in real-world situations (Tversky and Kahneman, 1992).

In the decision-making of CWWRB, the prevalence of objective uncertainty and complexity makes it difficult for construction workers to obtain complete information for optimal decisions. Additionally, as individuals with bounded rationality, factors such as cognitive characteristics, risk preferences, and empirical criteria further impede construction workers' ability to adhere to rational logic in practical decision-making. Faced with the above decision-making dilemmas, the explanatory power of traditional Cost-Benefit Theory appears insufficient. Given the advantages of Cumulative Prospect Theory in accurately depicting the decision-making under cognitive biases such as reference dependence, loss aversion, and probability weighting, this study combines Cumulative Prospect Theory and Cost-Benefit Theory to deeply investigate the decision-making process of CWWRB (Geng et al., 2023).

Integrating Cost-Benefit Theory with Cumulative Prospect Theory, considering the behavioral inertia and herd mentality of construction workers, the average expectation of behavioral decision-making on the previous day (AEP) is chosen as the reference point for gain and loss. *x*_*i*_ denotes the gain or loss of the risk event relative to the reference point, defined as the difference between the behavioral expectation and the reference point, yielding a sequence of gains and losses, as shown in Equation 2.


(2) $$\begin{array}{l} \begin{array}{c} {\text{x}}_{1}<{\text{x}}_{2}<\cdots <{\text{x}}_{\text{k}}<0\leq {\text{x}}_{k+1}<\cdots <{\text{x}}_{\text{T}} \end{array} \end{array}$$

The objective probability of *x*_*i*_ occurring is *p*_*i*_, and Tversky and Kahneman (1992) propose the corresponding weighting in Equation 3.


(3) $$\begin{array}{l} \begin{array}{c} \pi_{1}^{-}\;=\;\omega^{-}\left({\text{p}}_{\text{i}}\right)\\ \pi_{\text{i}}^{-}\;=\;\omega^{-}\left({\text{p}}_{1}+\cdots {\text{p}}_{i}\right)-\omega^{-}\left({\text{p}}_{1}+\cdots +{\text{p}}_{\text{i}-1}\right),\;{\text{x}}_{1}<{\text{x}}_{\text{i}}<0\\ \pi_{\text{i}}^{+}\;=\;\omega^{+}\left({\text{p}}_{\text{i}}+\cdots +{\text{p}}_{\text{T}}\right)-\omega^{+}\left({\text{p}}_{\text{i}+1}+\cdots +{\text{p}}_{\text{T}}\right),\;0\leq {\text{x}}_{\text{i}}<{\text{x}}_{\text{T}}\\ \pi_{1}^{+}\;=\;\omega^{+}\left({\text{p}}_{\text{T}}\right) \end{array} \end{array}$$

ω^+^(*p*) and ω^−^(*p*) are weighting functions for gain and loss scenarios, respectively, as defined by Equation 4 (Tversky and Kahneman, 1992). γ and δ in Equation 4 represent probability distortion parameters, which are set at 0.61 and 0.69, respectively (Tversky and Kahneman, 1992).


(4) $$\begin{array}{l} \begin{array}{c} \omega^{+}\left(\text{p}\right)\;=\;\frac{{\text{p}}^{\gamma }}{{\left[{\text{p}}^{\gamma }+{\left(1-\text{p}\right)}^{\gamma }\right]}^{\frac{1}{\gamma }}}\;\\ \omega^{-}\left(\text{p}\right)\;=\;\frac{{\text{p}}^{\delta }}{{\left[{\text{p}}^{\delta }+{\left(1-\text{p}\right)}^{\delta }\right]}^{\frac{1}{\delta }}} \end{array} \end{array}$$

Kahneman and Tversky (1979) propose the value function, which is illustrated in Equation 5.


(5) $$\begin{array}{l} \;V\left(t\right)=x^{\alpha },x\geq 0\\ V\left(t\right)=-\text{λ}\times {\left(-x\right)}^{\beta },x<0 \end{array}$$

In Equation 5, α and β represent the risk preferences of construction workers, typically set at 0.88, while λ reflects the importance of losses relative to gains of equivalent value, usually set at 2.25 (Tversky and Kahneman, 1992). According to Equation 5, the prospect value of CWWRB (PVRB) and the prospect value of construction workers' CWWNRB (PVNRB) can be calculated, and the difference in prospect value between CWWRB and CWWNRB (DPV) is computed as the basis for construction workers' decisions. A positive DPV represents CWWRB, while a negative DPV represents CWWNRB.

## 4 Empirical research

### 4.1 Data collection and variable assignment

Shenzhen is a pioneer in the construction of “No Waste City” in China (Ministry of Ecology and Environment of the People's Republic of China, 2022), with China Construction Eighth Engineering Division playing an exemplary role in green construction practices (China Construction Industry Association, 2017; China Association of Construction Enterprise Management, 2023). Therefore, this study selects the “Xinqiao Smart City” project in Shenzhen, undertaken by the aforementioned company, as the subject of empirical study (General Office of the People's Government of Shenzhen Municipality, 2022). The project, which utilizes a frame shear wall structure, spans approximately 243,000 square meters and has a contract value of 688 million yuan. The project department has developed a “Green Construction Plan,” setting a target of no more than 300 tons of construction waste per 10,000 square meters. To achieve this, measures such as rewards and penalties, training, and supervision have been introduced to promote CWWRB. Considering the phased progress goals of the case project, 120 days are selected as the simulation period for the model.

Data collection was completed through two-stage field interviews. The first stage focused on five on-site managers and aimed to understand waste management measures and the overall performance of construction workers. The second stage targeted 53 construction workers to explore the causation of CWWRB. The two-stage interview outlines and surveyed construction workers' demographics (Supplementary Table S1) are shown in Supplementary material. Following the approach of Ding et al. (2023), closed-ended survey data were processed using weighted averages, and open-ended responses were evaluated in conjunction with literature reviews, yielding the parameter results shown in Table 2. Notably, interview findings indicate that the project does not provide economic subsidies or rewards for CWWRB in actual construction. Additionally, some variables in this study require mathematical quantification, which will be detailed below.

**Table 2:** Assignment of partial variables and parameters.

**Description**	**Variables/ parameters**	**Values**
Work income of construction workers	WI	329.75
Average continuous daily working duration for construction workers	*n*	8.622
Growth rate of stress for construction workers	ν_*m*_	4.375%
Differences in the stress capacity of construction workers	ε	3
Negative incentive level	NIL	20
Probability that CWWRB or CWWNRB is detected by managers	MP	0.76
Initial probability that construction workers perform waste reduction behavior	P_0_	0.406
Probability of construction workers reminding others	PCWR	0.4
Impact of full effects of education and training on the probability of CWWRB	IFEET	1.64
Impact of incentive on the probability of CWWRB	IIP	1.279

#### 4.1.1 Variables of WI (work income of construction workers), WC (working cost), and AWC (additional working cost for CWWRB)

Construction workers devote considerable effort to ensure the timely completion of construction tasks to secure wages (Liang et al., 2018). Therefore, the average daily wage of construction workers is assigned to the variable WI. Meanwhile, this study defines the variable WC based on the fatigue level as defined in Equation 6 (Yang and Wang, 2021), in which 120 represents the conversion factor of the baseline fatigue degree and the monetary, with the values of other relevant parameters shown in Table 2. Interviews reveal that CWWRB requires an additional 18.77% in the working cost. Based on Equation 6, the value of WC for CWWRB and CWWNRB is 176.6 yuan and 209.7 yuan, respectively.


(6) $$\begin{array}{l} \begin{array}{c} \text{E}\;=\;120\overset{\text{n}}{\underset{\text{m}\;=\;1}{\prod }}\left(1+\nu_{\text{m}}\right)+\varepsilon \end{array} \end{array}$$

#### 4.1.2 Variable WETE (wasteful education and training effects of construction workers' CWWNRB)

Education and training can encourage CWWRB by compensating for construction workers' cognitive and experiential limitations in waste reduction (Li et al., 2022). Survey results show that the training interval for the project is 15 days. Due to CWWNRB, the wasteful effect of the jth training round on the ith day is described in Equation 7 (Yang and Wang, 2021).


(7) $$\begin{array}{l} \begin{array}{c} {\text{E}}_{\text{ij}}\;=\;\theta_{\text{j}}\times \left(1-0.56{\text{t}}^{0.06}\right) \end{array} \end{array}$$

**Figure 2:**
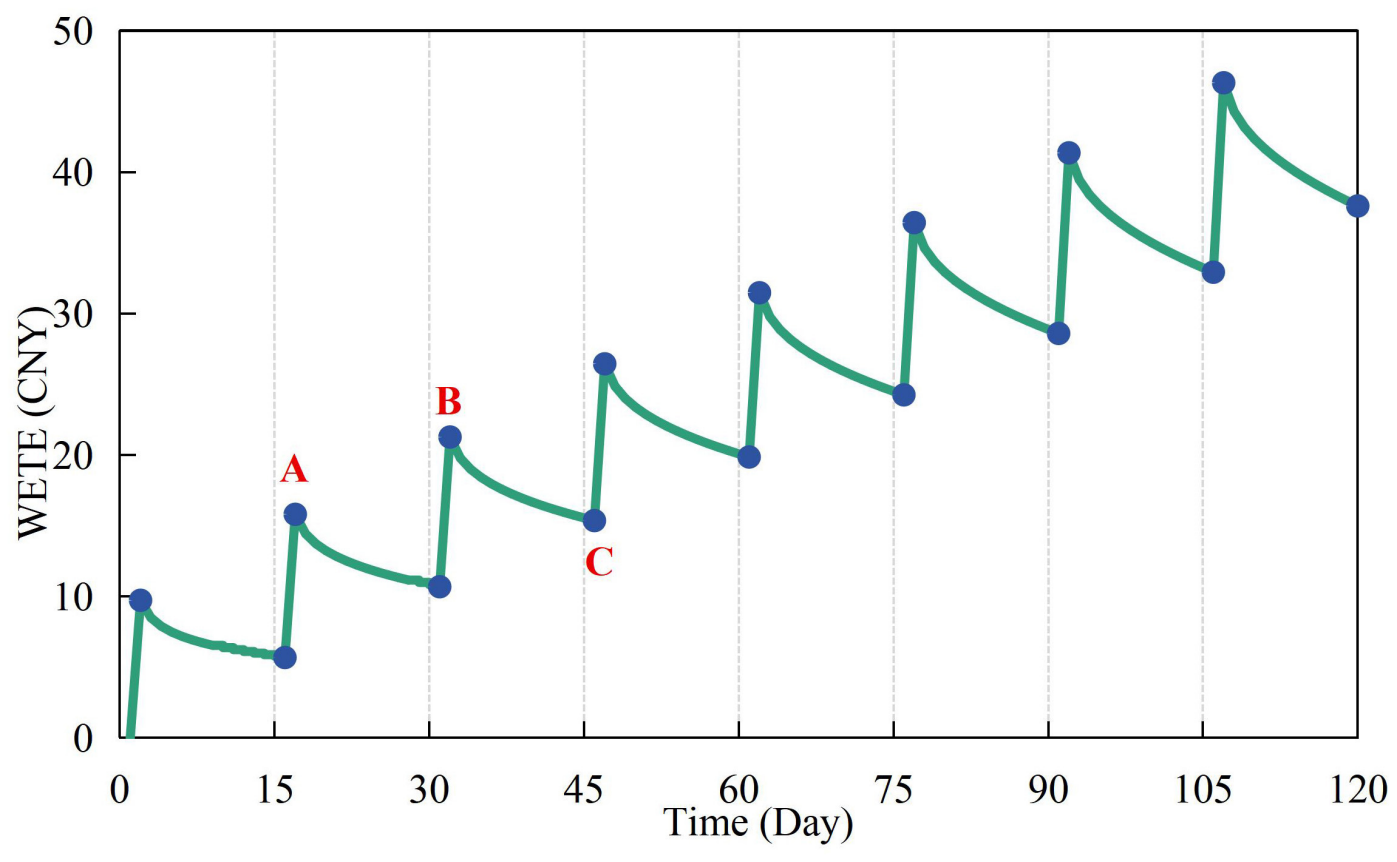
Values of the wasteful education and training effects of CWWNRB (WETE) during the simulation.

θ_*j*_ in Equation 7 is the initial effect value of the training, which is monetized in this study based on the average of the increase in behavioral expectations inspired by the complete education and training effect. 0.56 and 0.06 are the default parameter values of the Ebbinghaus forgetting curve, and t represents the number of hours since the end of the training (Yang and Wang, 2021). The cumulative wasteful effects of the first j rounds of training and the values of WETE were determined accordingly, as shown in Figure 2. Point A in Figure 2 marks the beginning of the wasteful effect of the second round of training, while points along segment BC represent the cumulative wasteful effect of the first three rounds of training on day i.

#### 4.1.3 Variable IMIP (impact of manager intervention on the probability of CWWRB)

**Figure 3:**
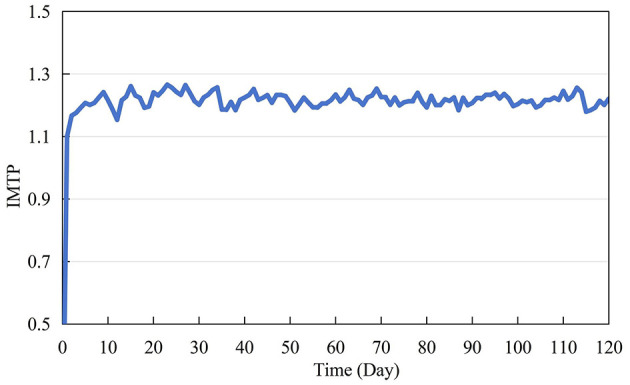
Values of the impact of manager intervention on the probability of CWWRB (IMIP) during the simulation.

The ratio of managers to construction workers is 1:10. Assuming that each manager randomly intervenes with a construction worker daily, and that construction workers initially choose to implement waste non-reduction behavior, construction workers randomly maintain waste reduction behavior for 1–4 days post-intervention. This study simulates the impact of 100 managers intervening in the decision-making of CWWRB to determine the value of IMIP, as shown in Figure 3. Figure 3 depicts that the value of IMIP initially rises overall and then fluctuates around the 1.2 level. The limited initial scope of manager interventions, coupled with the short-term persistence of construction workers' reactions to interventions, results in a continuous increase in IMIP values until the interventions are fully covered.

#### 4.1.4 Variable AWR (atmosphere for waste reduction)

Construction workers are influenced by a positive waste reduction atmosphere and gradually recognize the regulations for construction waste reduction management, thereby enhancing the willingness to reduce construction waste (Suciati et al., 2018; Paz and Delgado, 2020). This study quantifies AWR based on the efficiency of construction workers in complying with the reduction management regulations. The specific value of AWR is determined by drawing on the findings of Yuan et al. (2012) and the interviews, as detailed in Table 3. Table 3 shows that the value of AWR gradually increases from 0.2084 to 0.6267 as the project progresses, and the trend aligns with the conclusions of Yuan et al. (2012).

**Table 3:** Values of AWR the atmosphere for waste reduction (AWR) during the simulation.

**Day**	**AWR**	**Day**	**AWR**	**Day**	**AWR**
1	0.2084	45	0.4815	90	0.5664
5	0.2269	50	0.4988	95	0.5753
10	0.2499	55	0.5072	100	0.5845
15	0.2749	60	0.5155	105	0.5949
20	0.3036	65	0.5239	110	0.6056
25	0.3352	70	0.5324	115	0.6162
30	0.3692	75	0.5409	120	0.6267
35	0.4062	80	0.5493		
40	0.4466	85	0.5578		

#### 4.1.5 Variables of IPP (impact of production pressure on the probability of CWWRB) and PP (production pressure)

The extra time required by CWWRB will aggravate the production pressure of construction workers, especially under tight schedules, even leading to delays in construction progress (Al Nahyan et al., 2019). Furthermore, as the schedule advances, managers are more inclined to prioritize the production schedule, prompting construction workers to focus on production tasks under production pressure and managerial authorization, thus affecting the probability that construction workers perform waste reduction behavior (P) (Tam, 2008). Equation 8 is the assignment formula for variables IPP and PP, where *T*_0_ represents the number of days the project has been ongoing, and *T* is the total number of days for the project.


(8) $$IPP=1-PP\times \left(1-AWR\right)=1-\frac{T_{0}}{T}\times \left(1-AWR\right)$$

### 4.2 Model validation

Model validation is a crucial phase to determine that the model truly reflects the behavioral decisions of construction workers in the real world (Qudrat-Ullah and Seong, 2010). Therefore, extreme condition tests and sensitivity analysis were conducted.

#### 4.2.1 Extreme condition test

**Figure 4:**
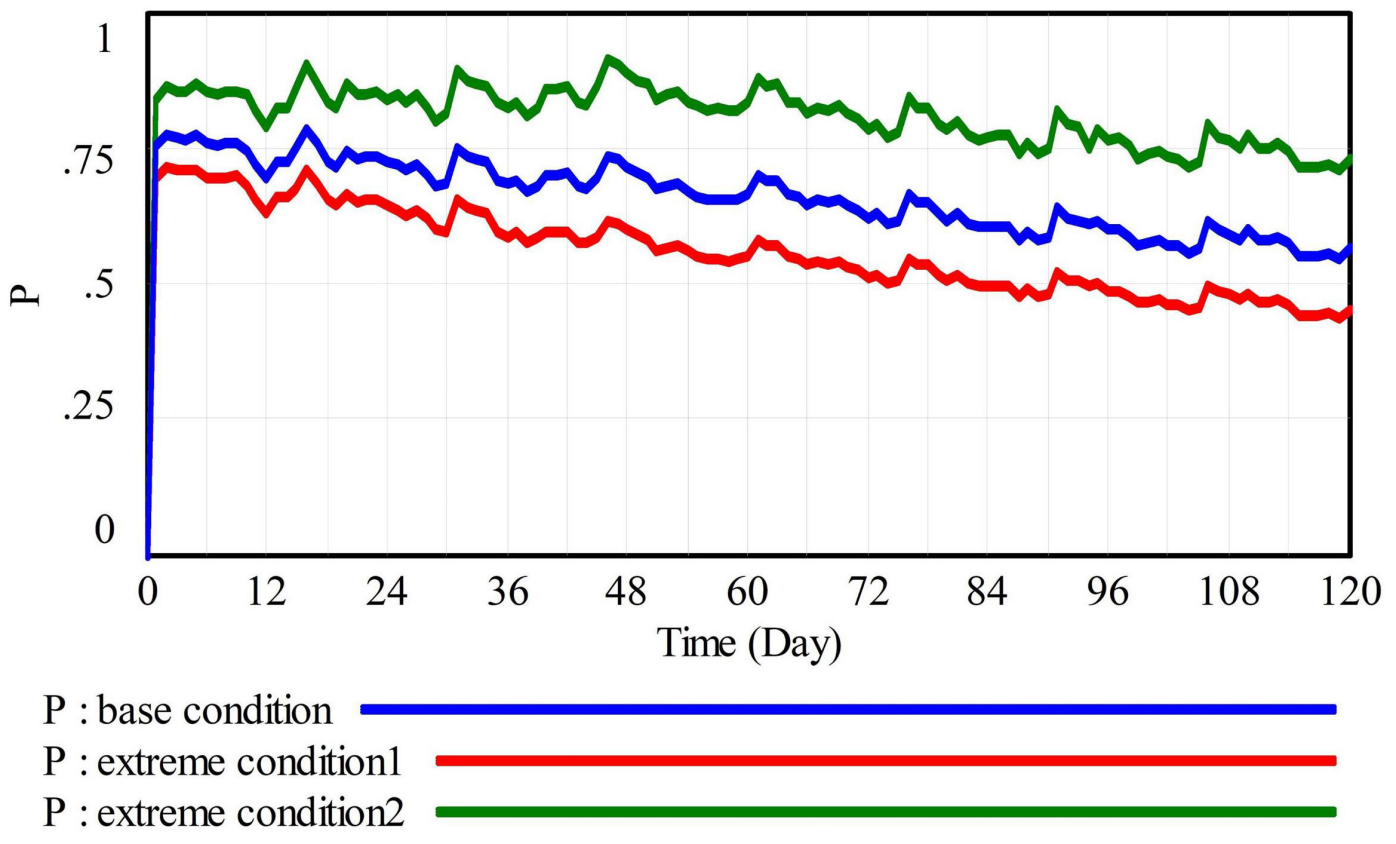
Results of extreme condition test.

Extreme condition test typically involves assigning extreme values to specific variables to observe whether system behaviors align with expectations, thereby assessing the credibility of the model under extreme conditions (Liu et al., 2023). This section uses the variable PCWR (Probability of construction workers reminding others), which ranges from 0 to 1, as an example. This section simulates the variations in the probability that construction workers perform waste reduction behavior (P) under extreme condition 1 (PCWR = 0), base condition (PCWR = 0.4), and extreme condition 2 (PCWR = 1) as depicted in Figure 4. Under extreme condition 1, where colleagues do not intervene in CWWNRB, P shows an overall declining trend, eventually falling below 0.5. Under extreme condition 2, colleagues remind CWWNRB, and construction workers tend to accept the reminder under a reduction atmosphere, which leads to an increase in P, showing a lower overall decrease than the other two conditions. Meanwhile, P under base condition lies between those of the two extreme conditions, displaying a similar trend. The above results confirm that the changes in P under extreme conditions align with expectations, indicating that the variable successfully passes the extreme condition test.

#### 4.2.2 Sensitivity analysis

**Figure 5:**
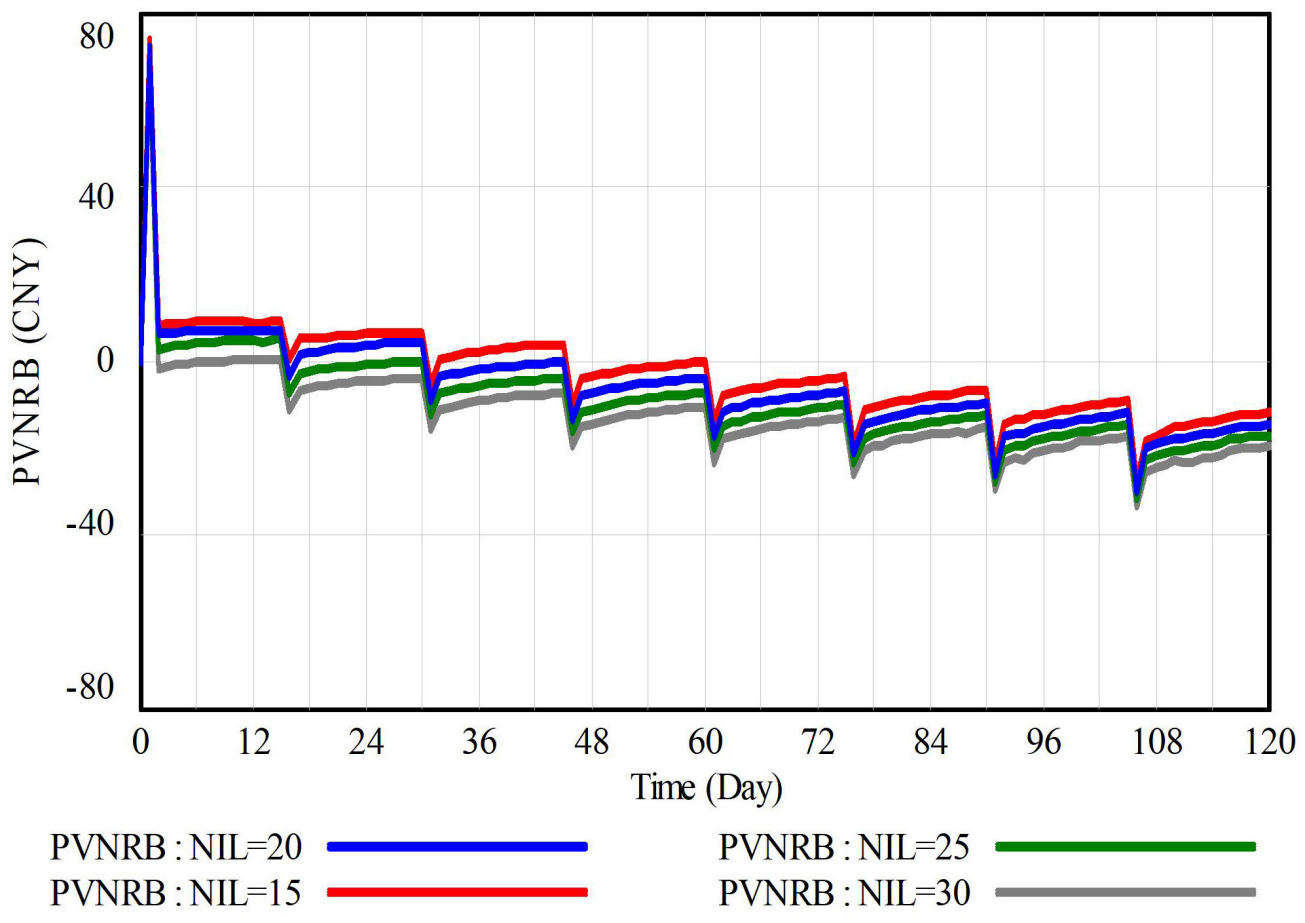
Results of sensitivity analysis.

Sensitivity analysis evaluates the model robustness by observing whether the model responds reasonably under minor fluctuations of variables (Forrester and Senge, 1980). Within the framework of Cumulative Prospect Theory, construction workers exhibit a high sensitivity to losses, with the impact of losses on psychological value expectations of construction workers significantly exceeding that of equal gains, making minor fluctuations in the negative incentive level (NIL) directly and significantly trigger the model response, thereby providing a more intuitive validation of the model's robustness. In contrast, other variables such as PIL and AWR also have a certain impact on the decision-making of construction workers, but their influence is relatively indirect or weaker, so they were not incorporated into the sensitivity analysis of this study. Based on the above reasons, this section focuses on the variable NIL, simulating its impact on the prospect value of construction workers' CWWNRB (PVNRB) under fluctuations of −25%, 0, +25%, and +50%, with values increasing from 15, 20, 25 to 30, as shown in Figure 5. The findings from Figure 5 indicate: (1) Negative incentives effectively suppress CWWNRB. Since construction workers are loss-sensitive individuals, an increase in NIL significantly reduces the estimated value of PVNRB, thus effectively constraining CWWNRB. (2) The trends in PVNRB under different scenarios show high consistency, demonstrating the stabilizing impact of NIL fluctuations on PVNRB. The impact of NIL fluctuations on the model is reasonable and stable, thus it can be concluded that the variable NIL passes the sensitivity test.

### 4.3 Analysis of simulation results for base run

**Figure 6:**
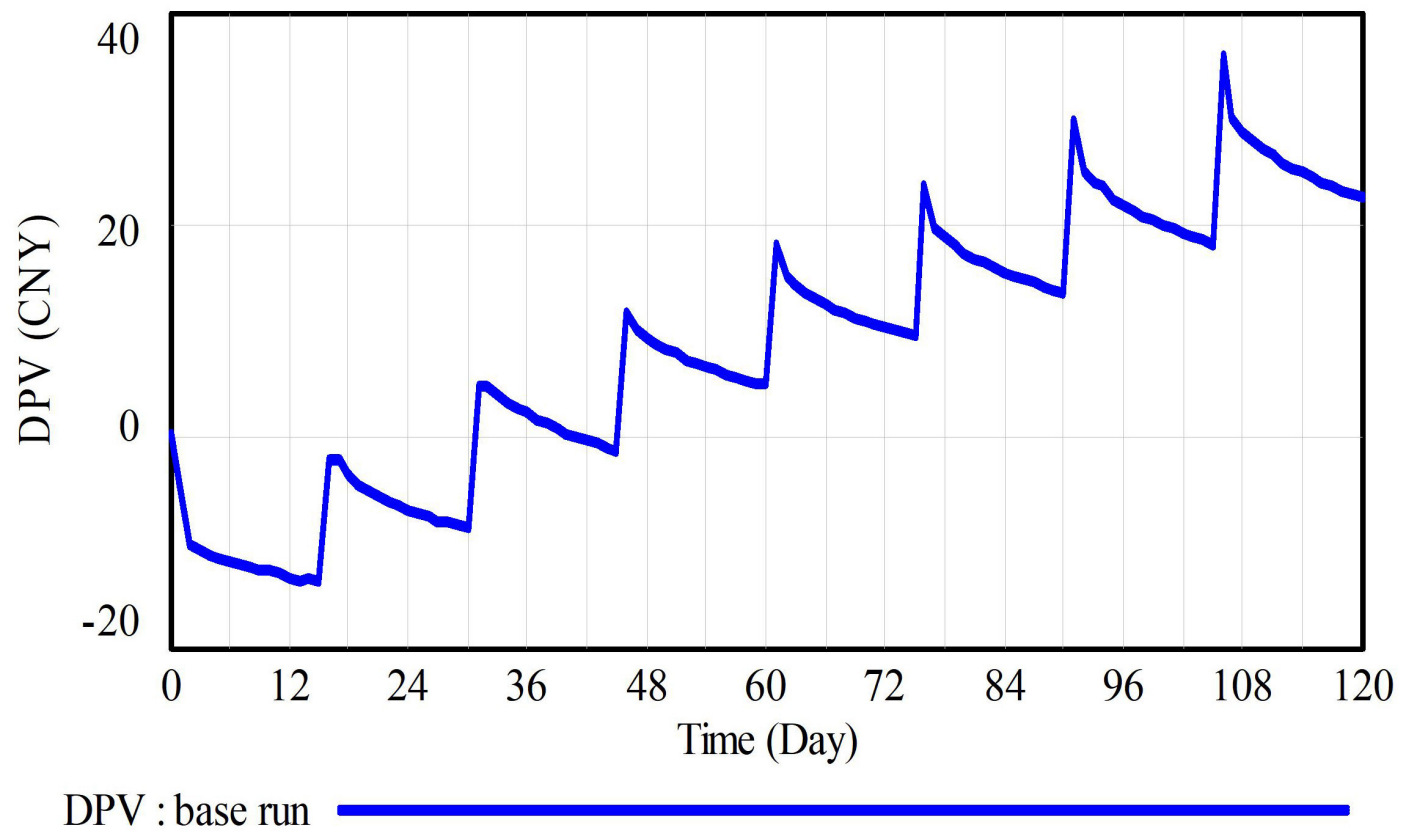
Results of base run.

This section, based on the field research data of the case project, simulates the dynamic changes in the difference in prospect value between CWWRB and CWWNRB (DPV) over 120 days to analyze construction workers' decisions influenced by self-interested motivations and cognitive biases. The simulation results are shown in Figure 6. Overall, the value of DPV shows a trend of decrease in the early stage followed by a periodic wave-like increase.

Within the first 15 days of the simulation, the value of DPV is negative and continues to decline, indicating the occurrence of CWWNRB. Due to the absence of a reference point on the first day, the loss-avoidant construction workers choose CWWNRB, which is less stressful, resulting in a negative DPV. Starting from the second day, construction workers adjust behaviors based on decision outcomes on the previous day, leading to a turning point in the DPV curve. Over time, the effects of education and training gradually diminish, weakening the suppressive influence of the wasteful education and training effects (WETE) and the remaining effects of education and training (REET) on CWWNRB, thereby causing a continuous decline in DPV at a lower rate.

From the 16th day onwards, DPV exhibits a periodic wave-like rise, turning positive for the first time on the 31st day and stabilizing in the positive range from the 46th day, reaching a peak on the 106th day. Construction workers are extremely sensitive to loss, resulting in the cumulative effect of WETE significantly reducing the prospect value of construction workers' CWWNRB (PVNRB), which is the key reason for the overall cyclical increase in DPV. Moreover, as the project progresses, the increasing production pressure (PP), combined with the weakening REET, leads to an initial rise followed by a fall in DPV within each wave cycle. Notably, the continuous optimization of waste reduction atmosphere gradually mitigates the declining trend in DPV.

### 4.4 Scenario analysis

The purpose of scenario analysis is to explore the specific effects of “Increasing the negative incentive level” and “Optimizing the atmosphere for waste reduction” on the decision-making of CWWRB. To this end, this section constructs two single-strategy scenarios (Scenario I and Scenario II) and one combined-strategy scenario (Scenario III), to observe the dynamic response of the difference in prospect value between CWWRB and CWWNRB (DPV) to adjustments in management measures.

#### 4.4.1 Scenario analysis of negative incentive strategy

**Figure 7:**
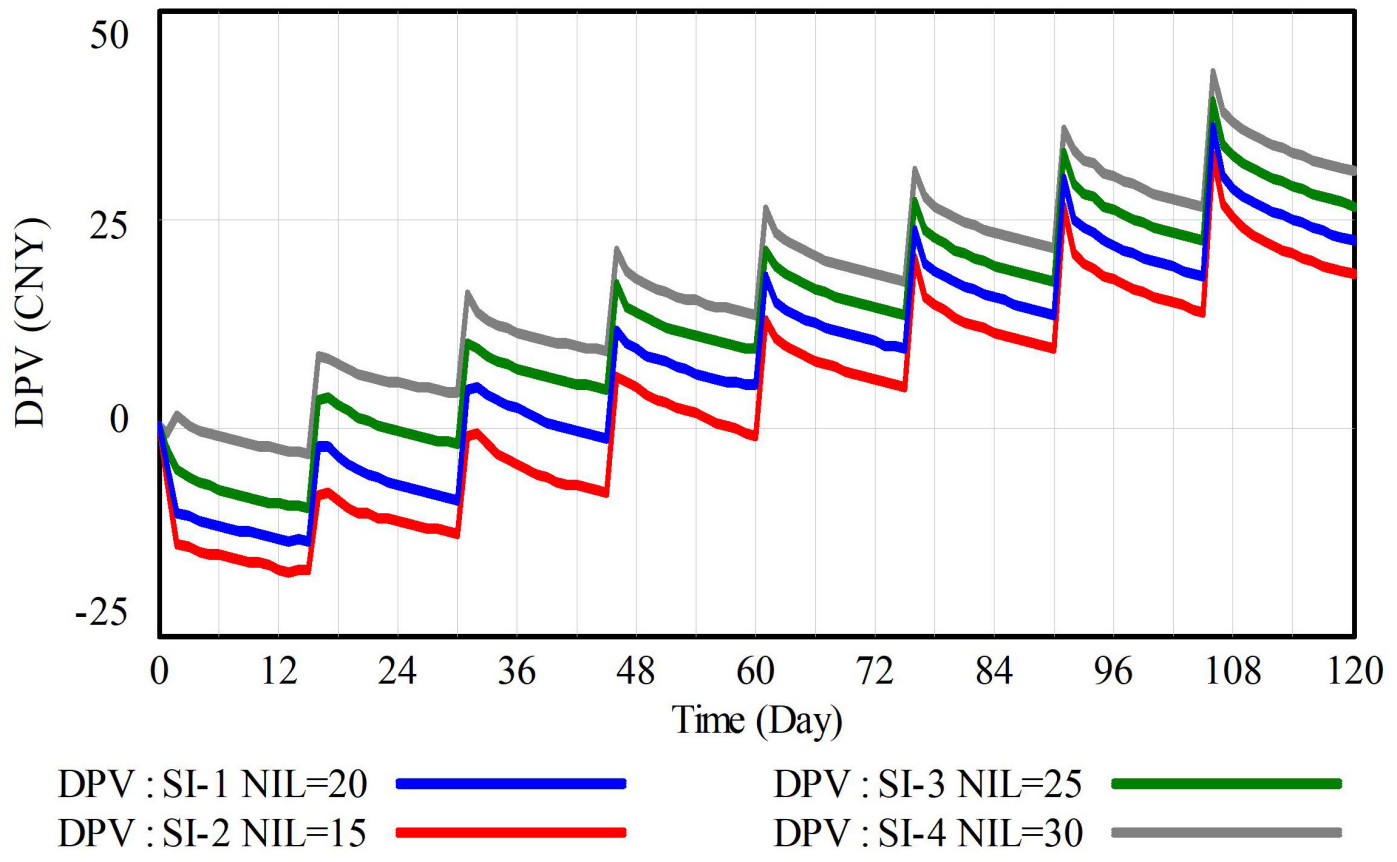
Simulation results of the difference in prospect value between CWWRB and CWWNRB (DPV) under scenario I.

Negative incentive level is defined as the penalty amount imposed on construction workers when CWWNRB is detected. Given the self-interested motivations and loss-sensitivity of construction workers, exploring how negative incentives affect the decision-making of CWWRB is crucial for refining incentive mechanisms and enhancing the efficiency of construction waste management (Tam, 2008; Yang et al., 2020). Therefore, in addition to the baseline scenario SI-1 (NIL = 20), this section posits three modified sub-scenarios where the negative incentive level (NIL) fluctuates by −25% (SI-2, NIL = 15), +25% (SI-3, NIL = 25), and +50% (SI-4, NIL = 30). The above scenarios are used to simulate the response of the difference in prospect value between CWWRB and CWWNRB (DPV) to changes in NIL, with results displayed in Figure 7.

Two conclusions can be drawn from Figure 7. First, increasing the negative incentive level significantly stimulates the decision-making of CWWRB. At the end of the simulation, DPV in scenarios SI-3 and SI-4 increased by 18.29% and 36.35%, respectively, compared to the baseline scenario SI-1. This increase is attributed to a significant reduction in the prospect value of construction workers' CWWNRB (PVNRB) estimated by loss-sensitive construction workers, leading to a rise in DPV and adjustments in decision-making. Second, since the 30th day, the impact of increased NIL on DPV exhibits a diminishing marginal effect, shown in Figure 7 as the narrowing distances between DPV curves with the increase in NIL, which is a result of construction workers' risk attitudes. Therefore, managers can resort to negative incentives to regulate the decision-making of CWWRB, but should be cautious of the potential reverse sentiment of construction workers triggered by excessive negative incentives (Wang et al., 2021).

#### 4.4.2 Scenario analysis of strategy for optimizing waste reduction atmosphere

**Figure 8:**
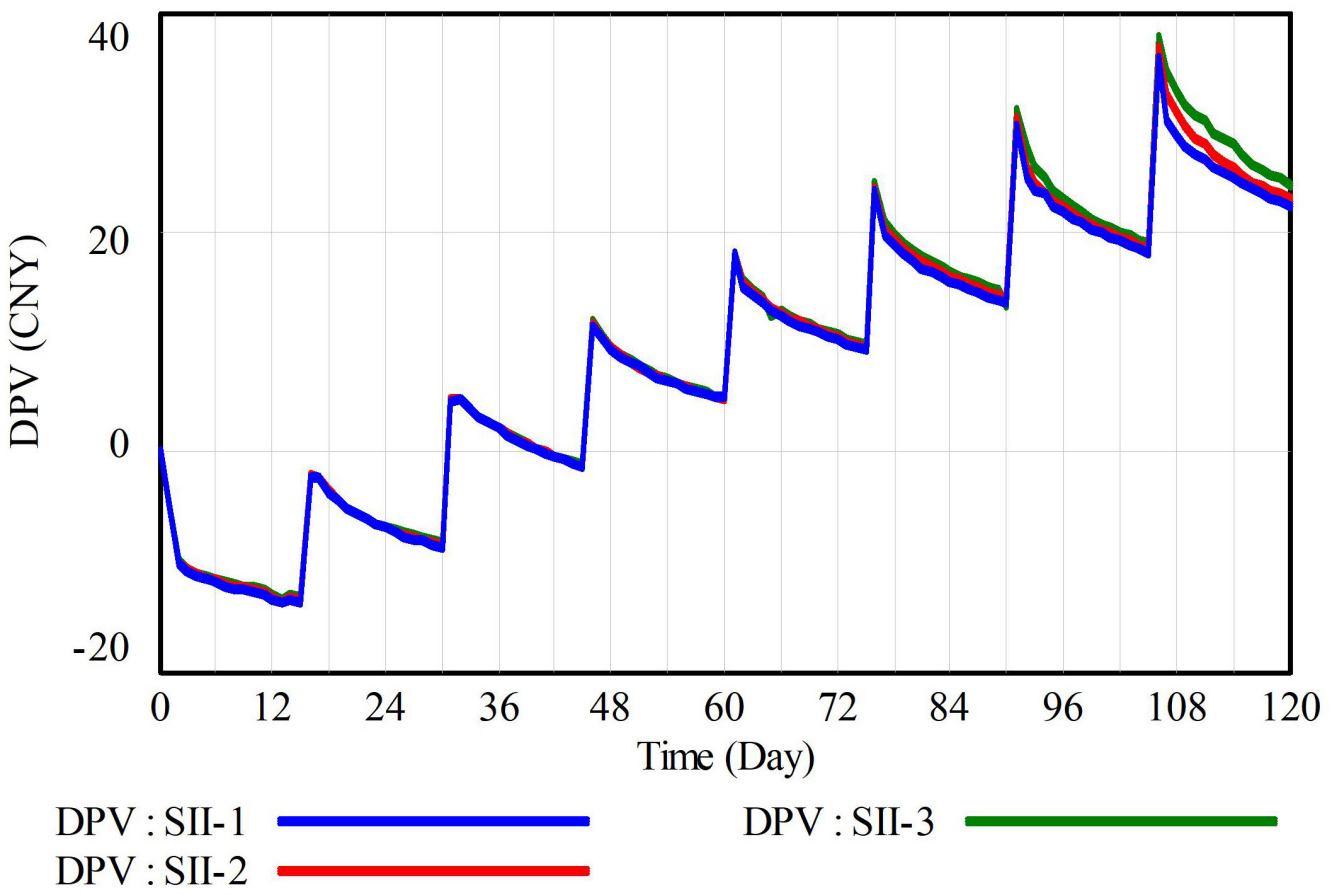
Simulation results of DPV under scenario II.

Working environment significantly influences individual behavioral choices (Essl et al., 2021), and a positive waste reduction atmosphere can effectively enhance the willingness of construction workers to implement waste reduction behavior (Yuan et al., 2018). Therefore, Scenario II sets a baseline scenario SII-1, along with modified scenarios SII-2 and SII-3, which, respectively, optimize by 20% and 40% in terms of waste reduction atmosphere, to reveal the specific impact on the decision-making of CWWRB. The simulation results are shown in Figure 8.

Figure 8 indicates that optimizing the atmosphere for waste reduction can promote the decision-making of CWWRB. It is worth noting that optimizing the waste reduction atmosphere exhibits a lagging effect on the difference in prospect value between CWWRB and CWWNRB (DPV), rooted in the time-accumulation effect of construction workers' recognition and familiarity with construction waste reduction management regulations. Therefore, it is recommended that contractors consistently implement measures such as on-site supervision, publicity and training, and incentives to maintain a positive waste reduction environment, thereby promoting long-term improvements in construction workers' behavior.

#### 4.4.3 Scenario analysis of combined-strategy

Scenario analysis of single-strategies has confirmed that both increasing negative incentives and optimizing the waste reduction atmosphere can promote the decision-making of CWWRB. This section aims to explore whether a combination of management measures can achieve better management outcomes. For this purpose, four sub-scenarios are designed, including the baseline scenario SIII-1, scenario SIII-2 with a 25% increase in the negative incentive level (NIL), scenario SIII-3 with a 40% optimization in the atmosphere for waste reduction (AWR), and scenario SIII-4 with both a 25% increase in NIL and a 40% optimization in AWR. In particular, SIII-2 and SIII-3 are single-strategy sub-scenarios, while SIII-4 is a combined-strategy sub-scenario.

Table 4 presents the changes in the difference in prospect value between CWWRB and CWWNRB (DPV) under single-strategy and combined-strategy. The “variation (%)” column in Table 4 represents the rate of change in DPV relative to the baseline scenario (SIII-1), reflecting the impact of management measure adjustments on construction workers' decisions. Scenario SIII-4 shows a significantly higher improvement in the value of DPV compared to other scenarios, at times even exceeding the sum of the effects of scenarios SIII-2 and SIII-3. This phenomenon suggests that combined management measures cover more dimensions of construction waste reduction management, effectively compensating for the diminishing marginal effect and lagging effect of single management measures, thereby providing insights for comprehensive optimization of management strategies.

**Table 4:** Comparative results of scenario analysis between single-strategy and combined-strategy.

**Day**	**SIII-1**	**SIII-2**		**SIII-3**		**SIII-4**	
	**DPV**	**DPV**	**Variation (%)**	**DPV**	**Variation (%)**	**DPV**	**Variation (%)**
20	−5.18	1.22	123.55	−5.09	1.74	1.26	124.32
40	0.21	5.70	2614.29	0.31	47.62	5.76	2642.86
60	4.79	9.28	93.74	4.87	1.67	9.53	98.96
80	17.29	21.25	22.90	18.30	5.84	22.49	30.08
100	19.84	23.99	20.92	20.73	4.49	24.94	25.71
120	22.42	26.52	18.47	24.16	7.76	27.92	24.53

## 5 Discussion

Construction workers play a pivotal role in the generation of construction waste, thus necessitating a thorough analysis of the decision-making of CWWRB (Teo and Loosemore, 2001). Existing studies have focused on analyzing the drivers of the decision-making of CWWRB (Bakshan et al., 2017), and the impacts of management measures on reduction targets and managers' decisions (Hao et al., 2019), yet discussions on the decision-making process are relatively scarce. Meanwhile, Yuan and Li (2018) and Yang and Wang (2021) emphasized that self-interests and cognitive biases significantly affect construction workers' decision-making, but do not elaborate on how these factors specifically affect the decision-making of CWWRB. Based on this, the study integrates Cost-Benefit Theory and Cumulative Prospect Theory, aiming to analyze the decision-making process of CWWRB influenced by self-interested motivations and cognitive biases, and explores the effect of management constraints on construction workers' decisions, thus contributing new insights to the field of construction waste research.

This article simulates the dynamic response of construction workers' decisions to different managerial measures. According to research by Hao et al. (2022), in the absence of incentive and mandatory measures, construction workers tend to overlook reduction targets. Accordingly, this study designs Scenario I, and finds that increasing negative incentive level can significantly promote the decision-making of CWWRB. The research conducted by Tam (2008) lends support to the findings of Scenario I, positing that a reward-penalty mechanism based on waste reduction performance can effectively motivate CWWRB. Yang et al. (2020) similarly emphasize the importance of combining incentive commitment with regulatory mechanisms. In contrast, this study further reveals the diminishing marginal effect of reinforcing negative incentives, which is rooted in the risk attitudes of construction workers.

The results for Scenario II indicate that optimizing the atmosphere for waste reduction is conducive to encouraging decision-making of CWWRB, aligning with the findings of Yuan et al. (2018). Yuan et al. (2012) make a similar argument that contractor's reduction management culture can lead construction workers to be more committed to reduction practices. Compared to existing studies, this study further reveals the lagging effect of optimizing waste reduction atmosphere on construction workers' decisions. Moreover, Scenario III suggests that combined measures can compensate for the diminishing marginal effect and lagging effect of single measures, achieving superior implementation effects and aligning with the conclusions by Jia et al. (2017).

Based on the results of scenario analysis, several suggestions to guide CWWRB are proposed: (1) Internalize CWWRB as a mandatory task for construction workers and improve incentive mechanisms, preventing potential reverse sentiment triggered by sole reliance on negative incentives. It is recommended that contractors promote a combination of negative incentives and on-site supervision, and implement graduated penalty measures to prevent habitual violations by construction workers. (2) Contractors should increase investment, strengthen training and supervision, establish demonstration posts and refine incentive mechanisms to create a long-term waste reduction atmosphere for construction workers. (3) Managers should implement the combined management measure of strengthening negative incentives and optimizing waste reduction atmosphere, thereby breaking through the limitations of single management measures. It is necessary to explore the optimal combination ratio of other management measures, thus providing a more comprehensive strategic framework for construction waste reduction management.

It is noteworthy that this study, using Shenzhen, China as a case study, investigated the impact of management measures on construction workers' decision-making of CWWRB and proposed corresponding management strategies. However, the generalizability of the findings may be significantly influenced by regional policies. Taking the “No Waste City” initiative in China as an example, Shenzhen, as one of the first pilot cities, has demonstrated a high level of policy implementation. Through a series of policy measures including financial subsidies, financial support, supervision and assessment, public participation, and education, Shenzhen has notably enhanced the awareness and willingness of both management personnel and construction workers regarding construction waste reduction, thereby creating favorable conditions for the effective implementation of management measures (Shenzhen Municipal People's Government, 2020). In contrast, in regions that have not responded to the “No Waste City” initiative or where policy enforcement is less stringent, the awareness and responsiveness of management personnel and construction workers to construction waste management measures are lower, making it difficult for management measures to effectively promote CWWRB, reducing the applicability of the findings of this study in these regions. Therefore, while this study has yielded valuable findings based on the Shenzhen case, caution should be exercised when generalizing these findings to other regions, taking into full consideration the differences in regional policies and other factors, and flexibly referencing the findings of this study.

## 6 Conclusion

Research on the decision-making of CWWRB is crucial for promoting the sustainable development of the construction industry. This study delves into the formation mechanism of the decision-making of CWWRB, and simulates the impact of different management constraints on construction workers' decisions based on case study, aiming to provide a reference for effective construction waste management. The key main are as follows:

(1) Model construction and validation

This study constructs a decision-making model for CWWRB based on Cost-Benefit Theory and Cumulative Prospect Theory, accounting for the coexistence of self-interested motivations and cognitive biases. The results of model validation show that the model is suitable for exploring the decision-making of CWWRB in real environments.

(2) Analysis of management scenarios

The simulation of various management scenarios leads to the following conclusions:

(2.1) Increasing the negative incentive level significantly promotes CWWRB, although it shows a diminishing marginal effect in the later stages.

(2.2) Optimization of the waste reduction atmosphere has a lagging guiding effect on the decision-making of CWWRB.

(2.3) Combined management measures can overcome the diminishing marginal effect and lagging impact of single management measures, thus achieving more effective management.

(3) Theoretical and practical significance

Theoretically, this study merges Cost-Benefit Theory and Cumulative Prospect Theory, and incorporates factors of self-interested motivations, cognitive biases, and management constraints, offering a new research perspective on the decision-making of CWWRB. This approach advances the updating of the theoretical framework of construction waste management. Practically, this study constructs a management framework that foster the participation of construction workers in waste reduction, providing guidance for construction waste reduction management in practice, and helping to address resource wastage and environmental pollution in the construction industry.

(4) Research limitations and prospects

This study, integrating Cost-Benefit Theory and Cumulative Prospect Theory, offers a new research perspective for understanding the decision-making mechanisms of CWWRB. However, there are issues worthy of further exploration. Firstly, the study primarily focuses on the individual level of construction workers, with limited exploration of the impact of team collaboration, social interactions, and other factors on decision-making. Future studies could employ Organizational Behavior Theory, Social Network Analysis, or Agent-Based Modeling to construct a more comprehensive decision-making model for CWWRB. Secondly, the data in this study originate from a single case, providing valuable insights for construction waste management but potentially limiting the generalizability of the findings. Future studies could enhance the generalizability of the findings by expanding data collection to include diverse regions and project types. Lastly, the management scenarios designed in this study do not encompass all possible management measures. Future studies could explore the impact of additional management measures on CWWRB based on existing studies and specific contexts, thereby providing more comprehensive and precise theoretical support for construction waste reduction management.

## Data Availability

The original contributions presented in the study are included in the article/Supplementary material, further inquiries can be directed to the corresponding authors.
