# Supplementary material for: Decision-making of construction workers' waste reduction behavior: a study based on Cost-Benefit Theory and Cumulative Prospect Theory
Source: Front Psychol. 2025 Mar 20;16:1557736. doi: 10.3389/fpsyg.2025.1557736 (PMC11967277; doi:10.3389/fpsyg.2025.1557736)
Supplement: Supplementary file 1 [file Presentation_1.pdf]

Supplementary Material

1 Informed Consent Statement for Participation in a Survey Study

Dear Mr./Ms. ___________________:

We cordially invite you to participate in a survey study aimed at understanding your work practices at construction sites. The goal of this survey is to improve future construction waste management practices and the working experience of construction workers. We seek your informed consent to participate in this survey. Please make your decision to participate after careful consideration. You may ask questions at any time during the consent discussion and request clarification on any aspect that you do not understand from the researcher.

We guarantee that the survey results will remain anonymous, and your decision to not participate will not result in any penalties or loss of benefits, nor will it affect your relationship with the researchers or their employers.

Before you agree to participate, the researcher will provide you with information about this study, including:

- The purpose of the research.
- The number of participants involved.
- What will happen during the study, including the time commitment and procedures you will undergo.
- Any potential risks or benefits.
- How your information will be protected and used in the future.
- How you will be informed about new findings that may affect you or your participation.
- Possible reasons for termination of your participation.

If you have any questions about your rights as a research participant, wish to discuss the survey study, have complaints or concerns, or need to obtain information or offer feedback, please contact:

Name: ____

Phone Number: ____

Email: ____

Consent Agreement

I have received an oral description of this study, including the information listed above. I agree to participate in this study and consent to the use of the survey results for publication in various forms of works.

Participant Name: ________________________ Contact Info: _______________

Participant Signature: _____________________ Date: _____________________

Representative Signature: __________________Date: _____________________
