# Supplementary material for: Decision-making of construction workers' waste reduction behavior: a study based on Cost-Benefit Theory and Cumulative Prospect Theory
Source: Front Psychol. 2025 Mar 20;16:1557736. doi: 10.3389/fpsyg.2025.1557736 (PMC11967277; doi:10.3389/fpsyg.2025.1557736)
Supplement: Supplementary file 2 [file Data_Sheet_1.pdf]

Supplementary Material

1 Interview for Outline Managers

1.1 Phase 1: Opening

We cordially invite you to participate in a research survey aimed at understanding the regulations for construction waste reduction management and the overall performance of construction workers in waste reduction. The data collected will be utilized for scientific research purposes. We guarantee that the survey results will remain anonymous, and that your choice to not participate will not cause any penalties or loss of benefits.

1.2 Phase 2: Interview Questions

We will gain insights into the management measures for reducing construction waste and the overall performance of construction workers through the following questions.

MQ1. How are education and training for construction waste reduction organized in the project?

MQ2. Does the project reward construction workers who implement construction waste reduction behavior? What is the form of these rewards?

MQ3. Does the project impose penalties on construction workers who violate the construction waste reduction regulations? What is the severity of these penalties?

MQ4. What is the ratio of construction workers to managers in the project?

MQ5. How do managers enforce reduction supervision and intervene with construction workers who do not reduce waste?

MQ6. What is the atmosphere for construction waste reduction on the construction site? How efficiently do construction workers comply with construction waste reduction regulations?

2 Interview Outline for Construction Workers

2.1 Phase 1: Opening

We sincerely invite you to participate in a survey study aimed at exploring the causes of construction workers' waste reduction behavior. The data collected will be used for scientific research. We guarantee that the survey results will remain anonymous, and choosing not to participate will not lead to any penalties or loss of benefits.

2.2 Phase 2: Interview Questions

We will collect demographic characteristics of the respondents, as well as detailed information related to the work of construction workers in waste reduction.

2.2.1 Question for demographic Information

DQ1. What is your gender?

DQ2. How long have you been working in construction labor?

DQ3. What is your age?

DQ4. What is your occupation in this project?

DQ5. What is your educational qualification?

2.2.2 Question for job-related Information

JQ1. What is your daily wage in RMB?

JQ1-1. What is your basic daily wage in RMB during the standard working hours?

JQ1-2. What is your hourly wage for overtime work outside the standard working hours?

JQ2. How many hours do you typically work each day?

JQ2-1. How many hours do you work overtime per day, apart from the standard working hours?

JQ3. How do you evaluate your daily work-related physical and psychological stress, and your ability to handle it? Does this change when you engage in construction waste reduction activities?

JQ3-1. What is the rate of stress growth in your work? (3% to 5%)

JQ3-2. How would you rate your own ability to handle stress? (0 to 10)

JQ3-3. How would you rate your colleagues' ability to handle stress? (0 to 10)

JQ3-4. How much additional physical and mental pressure will you bear if you implement construction waste reduction behavior?

JQ4. How likely are you to voluntarily invest additional time and effort in construction waste reduction without intervention?

JQ5. How frequently do you remind colleagues about construction waste reduction? How effective are their reminders to you?

JQ5-1. What is the likelihood of reminding colleagues to implement waste reduction behavior?

JQ5-2. How do you react to a colleague's reminder?

JQ6. What role can education and training play in your decision-making regarding construction waste reduction?

JQ7. What impact does the current economic incentive and penalty of the project have on your decision to reduce construction waste?

JQ8. How does the urgency of the project schedule affect your decision-making of construction waste reduction behavior?

JQ9. How does managerial supervision and intervention affect your decision-making?

JQ9-1. What is the likelihood that managers will notice your behavior?

JQ9-2: What actions will you take after being intervened by managers?

2.2.3 Supplementary Data

Demographic information and partial raw data from Interviews are shown in Supplementary Table S1 and Supplementary Table S2.

Supplementary Table S1. Demographic information of interviewed construction workers.

NO	Gender	Working experience	Age	Occupation	Educational qualification
1	Male	6 to 10 years	35 to 45 years old	Concrete Worker	Junior high school
2	Male	11 to 15 years	35 to 45 years old	Concrete Worker	Primary school
3	Male	Less than 5 years	25 to 35 years old	Concrete Worker	Junior high school
4	Male	Less than 5 years	35 to 45 years old	Concrete Worker	Junior high school
5	Male	11 to 15 years	Over 45 years old	Concrete Worker	Primary school
6	Male	6 to 10 years	35 to 45 years old	Concrete Worker	Junior high school
7	Male	Over 16 years	Over 45 years old	Concrete Worker	Primary school
8	Male	6 to 10 years	25 to 35 years old	Concrete Worker	Vocational and high school
9	Male	6 to 10 years	35 to 45 years old	Concrete Worker	Primary school
10	Male	6 to 10 years	35 to 45 years old	Concrete Worker	Primary school
11	Male	11 to 15 years	35 to 45 years old	Rebar Worker	Primary school
12	Male	11 to 15 years	Over 45 years old	Rebar Worker	Junior high school
13	Female	6 to 10 years	25 to 35 years old	Rebar Worker	Junior high school
14	Male	6 to 10 years	Over 45 years old	Rebar Worker	Primary school
15	Male	6 to 10 years	35 to 45 years old	Rebar Worker	Vocational and high school
16	Male	Less than 5 years	25 to 35 years old	Rebar Worker	College and above
17	Male	11 to 15 years	35 to 45 years old	Rebar Worker	Vocational and high school
18	Male	Less than 5 years	Under 25 years old	Rebar Worker	College and above
19	Female	Less than 5 years	35 to 45 years old	Carpenter	Primary school
20	Male	Over 16 years	35 to 45 years old	Carpenter	Junior high school
21	Male	Less than 5 years	25 to 35 years old	Carpenter	Vocational and high school
22	Male	11 to 15 years	Over 45 years old	Carpenter	Primary school
23	Female	11 to 15 years	35 to 45 years old	Carpenter	Junior high school
24	Male	6 to 10 years	35 to 45 years old	Carpenter	Primary school
25	Male	Less than 5 years	Under 25 years old	Carpenter	College and above
26	Male	11 to 15 years	35 to 45 years old	Carpenter	Junior high school
27	Male	Over 16 years	Over 45 years old	Carpenter	Primary school
28	Male	6 to 10 years	25 to 35 years old	Carpenter	Junior high school
29	Male	6 to 10 years	35 to 45 years old	Carpenter	Primary school
30	Male	Less than 5 years	Under 25 years old	Carpenter	Vocational and high school
31	Male	6 to 10 years	25 to 35 years old	Carpenter	College and above
32	Male	6 to 10 years	35 to 45 years old	Masonry Worker	Junior high school
33	Male	Over 16 years	Over 45 years old	Masonry Worker	Primary school
34	Male	6 to 10 years	25 to 35 years old	Masonry Worker	Junior high school
35	Male	6 to 10 years	25 to 35 years old	Masonry Worker	Junior high school

36	Male	Less than 5 years	25 to 35 years old	Masonry Worker	Vocational and high school
37	Male	6 to 10 years	Over 45 years old	Masonry Worker	Primary school
38	Male	Less than 5 years	Under 25 years old	Masonry Worker	Junior high school
39	Male	6 to 10 years	35 to 45 years old	Masonry Worker	Primary school
40	Female	6 to 10 years	Under 25 years old	Masonry Worker	Junior high school
41	Male	11 to 15 years	35 to 45 years old	Masonry Worker	Primary school
42	Male	Less than 5 years	Under 25 years old	Other Trades	Vocational and high school
43	Male	6 to 10 years	25 to 35 years old	Other Trades	College and above
44	Male	Over 16 years	35 to 45 years old	Other Trades	Primary school
45	Male	6 to 10 years	Under 25 years old	Other Trades	Vocational and high school
46	Male	11 to 15 years	35 to 45 years old	Other Trades	Primary school
47	Male	6 to 10 years	25 to 35 years old	Other Trades	College and above
48	Female	6 to 10 years	35 to 45 years old	Other Trades	Primary school
49	Male	11 to 15 years	Over 45 years old	Other Trades	Primary school
50	Female	6 to 10 years	25 to 35 years old	Other Trades	Junior high school
51	Male	Over 16 years	Over 45 years old	Other Trades	Junior high school
52	Male	Less than 5 years	Under 25 years old	Other Trades	Junior high school
53	Male	6 to 10 years	35 to 45 years old	Other Trades	Primary school

Supplementary Table S2. Partial raw data from interviews.

NO	JQ1-1	JQ1-2	JQ2-1	JQ3-1(%)	JQ3-2	JQ3-3	JQ3-4(%)	JQ4	JQ5-1	JQ6	JQ7	JQ9-1
1	280	50	1.0	3.8	4	8	10	0.3	0.3	1.5	1.3	0.7
2	290	55	0.5	4.3	5	9	30	0.3	0.5	1.6	1.4	0.75
3	260	50	1.0	4.8	6	10	10	0.7	0.3	1.5	1.3	0.5
4	240	45	1.0	5.0	3	5	33.3	0.3	0.5	1.7	1.1	0.9
5	300	55	0.5	4.5	7	4	5	0.6	0.5	1.5	1.5	0.7
6	290	55	1.0	4.6	6	5	10	0.3	0.2	1.3	1.3	0.9
7	320	60	0.5	3.8	5	8	20	0.5	0.5	1.7	1.5	0.7
8	290	55	0.5	4.5	3	6	25	0.3	0.3	1.5	1.3	0.6
11	370	70	0.5	3.5	1	7	10	0.5	0.3	1.5	1.4	0.8
12	400	75	0.5	4.1	4	8	20	0.6	0.8	1.5	1.5	0.7
13	270	55	0	5.0	4	8	45	0.2	0.1	1.9	1.3	0.9
14	350	65	0.5	4.7	8	10	30	0.25	0.15	1.7	1.1	0.8
15	330	60	0.5	4.8	5	5	20	0.3	0.5	1.7	1.33	0.7
16	300	50	1.0	4.3	5	10	20	0.8	0.7	1.95	1.5	0.55
17	320	65	0.5	4.0	6	8	10	0.3	0.3	1.5	1.3	0.7

18	280	50	0	4.9	5	4	20	0.3	0.5	1.6	1.2	0.9
19	270	50	1.0	4.6	3	6	25	0.35	0.5	1.9	1.7	0.7
20	340	65	0.5	4.0	9	8	15	0.6	0.3	1.3	1.1	0.9
21	280	50	1.0	4.8	8	10	10	0.3	0.3	1.7	1.3	0.7
22	300	60	0.5	3.9	5	7.5	10	0.65	0.5	1.5	1.3	0.7
23	290	55	1.0	4.6	6	7	15	0.5	0.5	1.7	1.55	0.9
24	290	55	0.5	4.2	6	10	20	0.5	0.3	1.6	1.1	0.75
25	300	50	0	5.0	7	5	30	0.15	0.3	1.9	1.2	0.9
26	310	60	0.5	4.5	4	10	25	0.4	0.5	1.7	1.3	0.7
27	330	60	0.5	3.4	7	5	20	0.3	0.5	1.7	1.6	0.7
28	290	55	1.0	4.0	6	10	15	0.3	0.5	1.8	1.3	0.9
29	300	55	1.0	4.7	8	5	20	0.5	0.3	1.75	1.3	0.7
30	280	50	1.0	4.4	4	8	10	0.4	0.3	1.7	1.2	0.88
31	290	55	0	4.0	3	8	10	0.4	0.2	1.5	1.35	0.8
32	300	55	0.5	4.3	3	6	25	0.3	0.25	1.6	1.2	0.9
33	350	65	0	4.9	10	8	10	0.6	0.6	1.8	1.2	0.75
34	300	60	0.5	5.0	3	7	36.7	0.1	0.3	1.7	1.05	0.8
35	280	50	1.0	4.4	6	4	20	0.4	0.3	1.6	1.3	0.9
36	250	45	1.0	4.3	9	4	10	0.9	0.65	2	1.4	0.9
37	270	50	1.0	4.2	1	5	10	0.3	0.5	1.5	1.2	0.8
38	250	45	1.0	3.7	8	7	15	0.25	0.33	1.8	1.1	0.7
39	280	50	0	4.7	4	7	25	0.1	0.4	1.8	1.1	0.85
40	290	55	0.5	5.0	10	7	20	0.5	0.6	1.9	1.2	0.8
41	310	60	0.5	4.5	5	10	20	0.2	0.3	1.3	1.2	0.9
42	250	45	0.5	4.0	8	6	16.7	0.33	0.3	1.4	1.45	0.7
43	300	55	1.0	4.3	4	7	25	0.6	0.6	1.33	1.3	0.9
44	360	70	0.5	4.1	5	9	20	0.6	0.3	1.6	1.4	0.7
45	260	50	1.0	3.6	7	5	5	0.7	0.5	1.7	1.1	0.6
46	330	60	1.0	4.5	8	6	33.3	0.5	0.3	1.5	1.25	0.8
47	310	50	0.5	4.2	4	9	15	0.3	0.45	1.85	1.2	0.75
48	300	55	1.0	4.9	5	7	25	0.4	0.3	1.6	1.3	0.5
49	350	65	0.5	4.2	6	4	15	0.5	0.5	1.7	1.2	0.7
50	250	45	1.0	3.9	4	8	20	0.4	0.6	1.75	1.2	0.9
51	320	60	0	5.0	2	4	30	0.3	0.25	1.5	1.15	0.7
52	200	40	0.5	4.2	4.5	6	10	0.3	0.45	1.2	1.2	0.85
53	270	60	0	4.4	8	6	10	0.3	0.4	1.6	1.1	0.5
